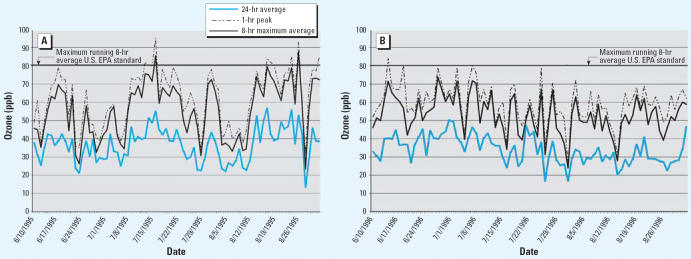# Errata

**Published:** 2006-08

In Figure 1 of the article by Triche et al. [Environ Health Perspect 114:911–916 (2006)], the 24-hr average and the 8-hr maximum average were labeled incorrectly. The corrected figure appears below:

On page 873 of the article by Sirivelu et al. [Environ Health Perspect 114:870–874 (2006)], two sentences were incorrect: “IL-1” was omitted from the first sentence and placed incorrectly in the second. The corrected sentences are as follows:

We have previously shown that NE levels in the AN are elevated after an immune stressor such as IL-1 (MohanKumar et al. 1998). AN has also been implicated in autonomic functions such as respiratory processing mediated by carotid body receptor (Banks and Harris 1988), suggesting that apart from the PVN, the AN also may be involved in stress-induced autonomic alterations.

*EHP* regrets the errors.

## Figures and Tables

**Figure:**